# Mr. Merriman, on Retroversion of the Womb

**Published:** 1806-11

**Authors:** Samuel Merriman

**Affiliations:** Curzon Street, May Fair


THE
Medical and Physical Journal.

VOL. XVI.] NOVEMBER, 1806. [NO. 93.

*Printed for R. PHILLIPS, by W. Thorne, Red Lion Court, Fleet Street, London.*

### *To the Editors of the Medical and Physical Journal.*

GENTLEMEN,

THE publication of unobserved facts and uncommon cases which occur in practice, can scarcely need an apology; for however great our attainments may be in the knowledge of diseases and their remedies, much yet remains to be learnt.

If the fact now communicated to you, respecting the uterus remaining in a state of retroversion to the complete term of gestation be not new, it is however unknown to the generality of medical practitioners; at least, most of those to whom I have mentioned the circumstances here related, acknowledged that they had never heard or read of any such instance.

The few slight observations which are offered upon extra-uterine fœtuses may perhaps require an apology; but as they are proposed with diffidence, I hope they will be received with candor, and that those who have more opportunities of information will investigate with greater accuracy a subject of much importance, and involved in much obscurity.

I am sensible that many imperfections may be discovered in the following pages, which time and more leisure might have corrected; but I was unwilling to delay the publication of a fact which is curious in itself, which adds something to the general stock of knowledge, and which may prove highly useful, should it ever be the means of preventing a painful, dangerous, and unnecessary operation. In thus acting I take the liberty of sheltering myself under the authority of Sydenham, who says, "Observationes quas collegi, sine morâ prælo mandandas curavi, ut si quid utile haberent, id statim publico cederet.

SAMUEL MERRIMAN.

*Curzon Street, May Fair, Sept.* 13, 1806.

Retroversion of the womb occurring during the early months of pregnancy, and occasioning a suppression of urine, is an accident not very unfrequent in practice. Before the time of the late Dr. William Hunter, to whose assiduity and acuteness of observation the science of Midwifery is much indebted, the nature and cause of this accident were much misunderstood, and many of those women in whom it occurred, lost their lives by the unskilful and injudicious treatment which was adopted.

A Practitioner of Midwifery in London, who had attended the lectures of the celebrated M. Gregoire of Paris, meeting with a case of retroversion of the womb in the year 1754, recollected the mention of a similar complaint by that Professor in his lectures on Midwifery; being however unable to relieve his patient, he requested the advice and assistance of Dr. Hunter; but their united endeavours were unavailing, and the patient died.

Dr. Hunter having obtained leave to open the body, invited a number of gentlemen of the faculty of medicine, to a public lecture which he gave, for the purpose of disseminating, as widely as possible, a knowledge of the disease, and of the method to be pursued for preventing a fatal termination. He afterwards, in consequence of two or three disastrous cases which occurred, published some remarks on this subject in the fourth volume of "Medical Observations and Inquiries, by a Society of Physicians," and caused an engraving to be made of the parts concerned, which is inserted in his very valuable Plates of the Anatomy of the Gravid Uterus. Thus the attention of practitioners of Midwifery was called to this peculiar disease; and since that time the cause and management of it have been so thoroughly investigated and defined, that few fatal cases now happen.* Recourse is had to the catheter in the early period of the retroversion, and the danger of the disease is speedily removed.

However, none of the authors who have written professedly on this subject, seem to have been aware that it was possible for the womb to continue in a state of retroversion to the full period of utero-gestation. It has been generally imagined that a suppression of urine, either partial or total, must be invariably produced by this situation of the womb, and that this suppression must be removed by art, otherwise

* Dr. Squire has related a case of rupture of the bladder from this cause, to which are subjoined some useful practical remarks.

See Medical Review and Magazine for 1801, vol. vi.

wise abortion, or a still more melancholy event, rupture of the bladder, would certainly ensue. Dr. Hunter, in his observations on this complaint, says, "Whenever the impregnated uterus is once thrown into that unnatural position, and continues in it some time, it will probably always remain so, unless reduced by art before it becomes so bulky as to be locked in the grasp of the pelvis; and in proportion as this process advances, the discharge both of urine and stools will become more difficult, *and at length both will be entirely suppressed.*"

This has been unquestionably the usual progress and termination of retroversion of the womb, when medical assistance has not been called in at the beginning of the complaint; but it is certain that a total suppression of urine has not always been the consequence of such an accident; and cases have occurred, in which the patients have attained the full period of utero-gestation, without any very uncommon or alarming symptoms, though a retroversion had taken place at an early period of pregnancy.*

A case of this kind, with the particulars of which I was well acquainted at the time, is recorded by Dr. Seguin Henry Jackson, in a useful little work, entitled, "Cautions to Women respecting the State of Pregnancy, &c." published in 1798, which I shall take the liberty of extracting. Dr. Jackson describes it in the following words.

"There is no instance on record, of a woman reaching the full period of gestation with a retroverted uterus. Such a case, however, I had an opportunity of seeing about two years ago, in company with Drs. Bland, Denman, Thynne, Merriman, and Croft. The situation of the patient at first appeared inexplicable, and she continued several days in labour, but the gradual efforts of Nature, at length, completed her delivery, by restoring the womb nearly to its natural situation. With great care she perfectly recovered, but the child, from the peculiarity of the case, as well as length of the labour, was still-born."

A case, similar in most particulars to the above,† has lately

---

* In addition to the cases hereafter mentioned, Dr. Thynne informs me that he not long ago met with a similar instance, which was terminated by the sole efforts of Nature.

† The principal difference between the two cases consisted in this, that Mrs. Wilkes, the woman mentioned by Dr. Jackson, suffered severely between the third and fourth months of her pregnancy from dysury and partial lately happened in a patient of my uncle, Dr. Merriman, which I had an opportunity of attending with him from the commencement; and as a publication of the particulars of it, will tend to render the knowledge of retroversions of the womb more complete, and as it may become of much practical utility, I have undertaken to give a detail of the symptoms and progress of the labor, and shall add a few observations arising from a consideration of the circumstances of the case.

Mrs. F****, of Welbeck Street, Cavendish Square, became pregnant of her first child, about September 1805. She did not appear to suffer more than most other women, during her pregnancy, except that for some of the last months she was troubled with difficulty of parting with her urine, and considerable pain in the act of passing it; yet her sufferings in this particular were not so great as to induce her to consult her accoucheur upon the subject, and at no period did she experience a total suppression of urine, nor can she recollect having ever retained it long enough to occasion any considerable inconvenience. When about five months advanced in her pregnancy, she was much terrified and affected at hearing of the sudden death of an aunt; which, as she expresses it, seemed to turn her whole inside *upside down,* and to this she imputes the alteration in the situation of the womb, which she was given to understand, either then or at some other time, took place;† this sudden surprise and fright seemed to her at least the most rational mode of accounting for it.

She partial suppression of urine, yet was enabled, without having recourse to the catheter, to pass enough to preserve the bladder from injury; and towards the conclusion of her pregnancy was relieved from much of this inconvenience, probably by the parts adapting themselves to the situation she was in; whereas the subject of the following case never felt any tendency to suppression of urine, nor suffered pain in parting with it, till the latter months of her pregnancy.

It will not be improper to add, that Mrs. Wilkes, in a subsequent pregnancy, by paying constant attention to the timely evacuation of the bladder, was preserved from the same kind of accident, and at the end of her reckoning was delivered of a living child, by Dr. Merriman, who found the uterus free from disease, and in its proper situation. Some months afterwards Mrs. Wilkes was attacked with symptoms of phthisis pulmonalis, and died consumptive.

† It is worthy of remark that in many cases of retroverted womb, the cause of the accident is attributed to some sudden alarm or fright, yet it has been ascertained, by the strictest inquiry and investigation, that over distension of the bladder is almost constantly the sole cause. See the very excellent chapter on this subject in Dr. Denman's Introduction to the Practice of Midwifery.

She was taken with symptoms of labor, viz. a serous discharge from the vagina, and strong pains recurring at distinct intervals, on Monday, June 16, 1806. These pains became so distressing in the course of that day, that it was thought necessary to make an examination of the state of the os uteri, and progress of the supposed labor; but upon such examination, no os uteri could be felt, the passage of the finger towards the os sacrum being prevented, by the attachment of the posterior part of the vagina, to a large semi-globular substance, which occupied the whole of the vagina, descending through the pelvis, and bearing down gradually towards the perinæum. This hard substance felt exactly similar to the womb enveloping the head of the child in cases of pendulous belly, where the os uteri, close and rigid, is thrown very high up in the vagina backwards, pressing against the upper part of the os sacrum, and the lower vertebræ of the loins. But there was this material difference, that in all those cases of pendulous belly, the finger can be carried up backwards to its utmost extent, though perhaps not far enough to reach the os uteri; whereas, in this case, the finger could not be carried more than an inch within the vagina, towards the sacrum, before it encountered a resistance, (like what the French call a *cul de sac*) arising from the firm attachment of the vagina to the posterior part of the uterus, as above stated.

By introducing a finger into the rectum, this substance could be more distinctly traced, presenting the idea of the fundus uteri containing the head or nates of a child, and fallen down between the vagina and rectum; while forwards, betwixt this substance and the ossa pubis, the finger could be carried up, though with difficulty, an unlimited length, but without discovering any traces of the os uteri.

Dr. Merriman, who had been engaged in attending, and indeed had delivered the other woman whose case is before spoken of, was well assured in his own mind that this was of a similar nature; and as there were at present no alarming symptoms, he left her to the efforts which Nature seemed to be making in her favor.

The patient, in the mean time, and during the whole of Tuesday the 17th, was tormented with very frequent and excruciating pains, which made no apparent impression on the uterus, except bringing down this semi-globular substance nearer to the perinæum. Though every method was adopted to keep her in a cool and temperate state, the severity of the pains at length brought on a considerable degree of fever and delirium, and she was seized with violent convulsion fits, to the great alarm of her relations and attendants.

Dr. Merriman, on being now summoned to her again, found her in the alarming state above described. He immediately directed that she should be bled to the amount of ten or twelve ounces, that a stimulating clyster should be injected, and when that had operated, that she should take an anodyne draught. The fever being by these means stopped *in limine*, gave no further alarm, the convulsions ceased, and she became in a few hours perfectly rational; the pains likewise diminished both in frequency and violence, and at intervals she enjoyed several hours sleep.

Under a plan of strict and regular attention, she continued in nearly the same state two or three days, during which time she passed her urine and fæces frequently, and without difficulty. The pains were regular and distinct, but much less severe than they had been, resembling more the common preparatory pains of labor; yet no sensible alteration was produced in the situation of the womb and its contents. Even on Friday, June 20, four days after the access of labor, it was impossible to carry the finger backwards within the vagina towards the sacrum, but it was, involuntarily as it were, directed by the uterine tumor towards the ossa pubis; yet nothing like the os uteri could be distinguished by the touch, notwithstanding the most particular and careful examination; however, upon withdrawing the finger, a mucous discharge tinged with blood was perceived upon it, which furnished a convincing argument that the os uteri was situated in that direction.

The idea which we entertained of the nature of this case, had been explained to the husband and other friends of the patient; they were told that very little chance remained of saving the life of the child, (indeed she had not once felt it move since the convulsions) but that we had good grounds to hope that the labor would terminate favorably in regard to the mother at least, and consequently that in her present state we should deem it highly injudicious, to attempt any artificial means to procure an exit for the child.

While this labor was thus slowly proceeding, several opportunities occurred of conversing with professional gentlemen respecting it, and among others with Doctor Denman, of whose great experience and high reputation, no practitioner of midwifery can be ignorant. From the description which was given to him, he seemed inclined to to consider this as a case of extra-uterine fœtus fallen down with the head between the vagina and rectum; yet did not appear so confident of his opinion as to venture to propose any operation for the release of the child; on the contrary, he approved of delay, as there was now no alarm from the fever or convulsions, and recommended that we should wait still longer, in hopes that Nature would point out some safe way for the patient's relief. Dr. Denman very kindly offered his assistance in this extraordinary case, if any circumstances should occur to render it advisable; and this offer being readily accepted, he accordingly visited the patient.

One circumstance seemed strongly to support the opinion that this was an extra-uterine fœtus. It was this; two tumours were clearly distinguished by the application of the hand to the part, in the left hypogastric region, apparently covered only by the external integuments of the abdomen. These tumors were supposed to be the head and shoulders of the child, and they appeared to lie so very near to the surface, that one could hardly believe that the parietes of even a remarkably thin uterus could be interposed between the integuments and the child's head, yet we had reason to think, from the feel of the uterus through the rectum, that the womb was there at least of the usual thickness.

On Saturday, June 21, the sixth day after the commencement of the labor, a considerable alteration was discovered in the nature of her pains, and in the situation of the hard semi-globular substance above-described. The pains pressed the uterus closer against the ossa pubis, between which and the body of the uterus there was evidently to be felt a thick fleshy substance of a flattened form, descending gradually by the effort of the pains into the vagina, at the same time that the tumor formed by the fundus uteri, which had been contiguous to and pressing on the perinæum, began to recede.

Dr. Denman being informed by a note of this change in the situation of affairs, was so obliging as to come to satisfy himself, and give his opinion of this unusual case. The flattened fleshy substance between the ossa pubis and the uterus felt by this time, as if it were the empty urinary bladder forced down in a retroverted state by the pressure of the cervix uteri gradually twisting itself round, and recovering its proper situation in the pelvis, though even at this time the os uteri could not be reached.

In order to ascertain the truth of this suspicion, Doctor

Denman proposed that an attempt should be made to introduce a catheter into the bladder, which was accordingly done. It passed almost in a straight line without any difficulty, and drew off about a tea-cupfull of straw-coloured urine, thereby refuting the conjecture we had formed, and ascertaining the sound state of the bladder and urethra.

In a few hours more, by the increased action of the uterine pains, the womb was gradually restored to its proper position, and the os uteri could now be distinctly felt, coming down from a considerable height above the pubes, and the child's head behind it.

As the flattened fleshy substance just mentioned came more within reach, it was perceptibly distended with some fluid by every pain, and as it approached the os externum, resembled to the touch, the membranes distended with the liquor amnii, but those membranes much thicker than in the natural state. Shortly after some of the bones of the cranium became loosened and forced down into this bag or pouch, and clearly evinced its being only the scalp of a dead and putrid child, and that its distension was owing to the contents of the cranium being forced into it by the pressure of the pains.

The death of the child being now rendered certain, Dr. Merriman determined upon making an opening through this distended scalp, in order to evacuate its contents, which he received in a bason, nearly to the amount of a pint, and which consisted entirely of grumous blood, and the brains of the putrid fœtus. Having now a good purchase by means of the scalp, he proceeded to assist during a pain in extracting the collapsed head, which passed easily through the vagina, and no further obstacle was presented to the delivery; the placenta, which was detached by the last pain, followed almost immediately.

Thus happily terminated a labor which could not at first be contemplated without much apprehension for the result. The patient had afterwards the most favorable symptoms, and recovered perfectly in less than a fortnight, but lost the cuticle of her whole body, which peeled off, in like manner as it does after violent and dangerous fevers.

The position of the womb here described is very rarely met with at so late a period of pregnancy, or at least has been rarely spoken of by authors; and those who have incidentally mentioned it, give no reason to think that they were sensible of the real nature of the case. Deventer, in in his chapter on the Obliquities of the Womb, describes the os uteri pressed against the ossa pubis as a situation occasionally found, but appears not at all aware, that the fundus uteri was in such cases thrown down between the rectum and the vagina, yet it is evident from the words he employs,* that such must be the position; for how could the passage of the finger towards the os sacrum be impeded, or a substance similar to the head of a child be felt in the vagina, whilst the os uteri was close to the symphysis of the pubes, unless the uterus were retroverted? Or what except this position of the womb should occasion the very great danger both to the mother and child which he laments? The mere pressure of the os uteri against the pubes, independent of a retroversion, would be removed without much difficulty by the action of the pains, and no particular danger or alarming symptoms could be expected to attend such a labor, though it might prove tedious; but Deventer speaks of the situation of the womb as most perilous, in which both mother and child often lose their lives.† It is apparent therefore that he was not conscious of all the circumstances of the position he describes, and we cannot wonder that the methods of relieving it, which he recommends, should be injudicious, and probably impracticable. It may perhaps after all be questioned, whether Deventer did not write on this subject from theory rather than from actual observation.

A case of uterus remaining retroverted to the complete period

---

* In hoc pravo uteri sitû, obstetrix probe attenta os uteri aut omnino non, aut parum saltem tangere poterit, nisi jam late satis pateat, atque tum adhuc aliquam saltem circuli partem attingere licebit. . . . . Obstetrix digitos posticam versus ad intestinum rectum intrudens nil nisi *sacculum clausum* offendit, et parum validius premens præ inscitia facile sibi persuadet, *infantis se capitulum sentire*, non dijudicans, utero adhuc tectum esse, ac se frustra ejus descensum præstolari: Obstetrix sagax in hoc rerum statu *proxime cervicem vesicæ sentiet oram aliquam lunatam*, quæ oris uteri est, quod si digitis illac penetraverit, etiam capitis partem duram, globosam, lævemque sive verticis aperturam sentiet, unde certo colligere potest, infantem pariter cum utero spinæ dorsi nimis apprimi.

Deventer de Partu Difficili ex Utero Obliquato.

† Non raro parturientem biduum triduumve, quin et quatriduum laborare, neque enim partum eniti contingit, et quod plus est, primis nonnunquam diebus dolores maxime vigent, ita ut mulier irritis defatigata laboribus infanti animam inscienter extinguat. . . . . Denique nullo fere aut languido doloris impulsu infans mortuus sponte quasi excluditur, sed singulari Dei providentia id fit ad conservationem matris quæ alias plerumque *simul occumbit, et moribunda sæpe fœtum edit exanimem.*

Deventer de Utero obliquato.

period of gestation has, if I mistake not, been described by the late Dr. Charles Kelly, in vol. iii. of Medical Observations and Inquiries, under a supposition that it was a case of extra-uterine fœtus. Many of the circumstances attending it, lead to an idea that this was in reality a retroversion of the womb terminating in a rupture of that organ.

Dr. Kelly states, that he was desired by Dr. Crawford to visit a poor woman who had then been about seven days in labor; on examining the situation of the uterus, &c. the following circumstances were observed: "On passing a finger into the vagina the head of a child was plainly felt, and so far advanced into the pelvis, that it seemed to require but two or three pains to bring it into the world. Yet although the head was so far advanced into the pelvis, as to be but the length of a finger-joint from the fourchette, the os tincæ was situated close to the symphysis of the pubes, and so high up, that it was very difficult to reach it, and so far as this difficult access would permit a judgment to be formed of the state of this part, it seemed to be not at all dilated."

"No circumstance so strongly indicated the head being in this situation, (between the vagina and rectum) as that of not being able to pass a finger between the tumor made by the head and the back part of the vagina; for in every natural case, where the head is so far advanced into the pelvis as in the present, even though the os uteri be not dilated, there is always a vacant space between the tumor made by the head and the back part of the vagina; but here there was none."

The foregoing extracts detail circumstances coinciding in so many particulars with such as occurred in Mrs. F's case, as lead to the conjecture that they both originated from the same cause, namely, retroversion of the womb.

It may however be objected against this opinion, that upon opening the body, the fœtus was discovered without the womb, lying between the vagina and rectum, and no mention is made of the womb being ruptured; but this objection cannot have much weight, for Dr. Kelly laments the haste with which he and his friend were obliged to make the examination, which he acknowledges prevented them from doing it "with the deliberation and accuracy they could have wished." It is therefore by no means improbable that they might overlook a rupture of the womb, through which the child escaped into the cavity of the abdomen, especially as their "hurry and confusion from being being so disagreeably circumstanced, prevented them from examining the appendages of the uterus."

Other cases have been recorded, which were probably of a similar nature, and misunderstood by the attending practitioners.

I think it not unlikely, that some of those cases which are to be found in Smellie's, and other collections, where the os tincæ is described as grown together and impervious, were actually retroversions of the womb; in these the supposition of a coalesced state of the os uteri has occasioned an artificial aperture to be made within the vagina into the uterus, generally I fear with fatal consequences to the mother.

The late Dr. Colin Mackenzie, in a letter to Mr. Perfect, mentions a case very similar to that published by Doctor Kelly, and proposes the same mode of relieving the patient, namely, by means of an incision through the posterior part of the vagina. This likewise was probably a case of retroversion of the womb.

Dr. Mackenzie thus speaks of it. "I knew an instance of a child found without the uterus in the abdomen of its mother. The pains came on and a midwife was employed; this woman finding an enlargement in the vagina, mistook that for the membranes, which she attempted to break through by repeatedly scratching it with her nails, in which she succeeded so far as to evacaute the waters; however, the birth of the child being still retarded, a man-midwife was procured, but to no purpose, the woman growing worse and worse, till at length she died. Now, had I been present, I cannot help flattering myself, that I might in all probability have saved both mother and child; for I believe I should have ventured to make an incision through that part of the vagina, which was thrust forward by the propelling force; and perhaps by that means might have delivered the child."

Perfect's Cases in Midwifery, vol. ii. p. 171.

Since the above was written, I have met with a French author, M. Lebas, Lecturer on Midwifery at Paris, who gives the first intimation I have seen, that the uterus may remain in a state of retroversion to a late period of pregnancy; and he supposes that such a situation of the womb must necessarily terminate in a rupture of that viscus; M. Lebas looks upon the death of the woman as an event almost inevitable, and that the child can only be saved by by an opening made into the abdomen of the mother,* but he enters into no details, reserving what he has further to take notice of, for a larger work which he announces as ready for publication, intituled "Traité complet d'Accouchemens;" this, if ever published, I have not been able to procure.

If M. Lebas be an author whose authority can be relied upon, his opinion strengthens the conjecture, that the cases related by Dr. Kelly and Dr. Mackenzie were really retroversions of the womb, ruptured either by the force of the pains or by the unskilful interference of the midwives.

There is likewise another class of cases recorded by writers on the subject of Midwifery, which may be supposed to have occurred in consequence of a retroverted state of the womb. I allude to those cases of extra-uterine fœtuses discharged per anum, or through an ulcerated opening in the vagina, after having been carried, sometimes for many years, in the abdomen of the mother. Thomas Bartholine in his book, "de insolitis humani Partus Viis," has collected several of this kind, which appear to have been retained in the uterus beyond the proper period, and were afterwards evacuated by means of a communication into the rectum.† And Dr. Garthshore, in his "Observations on extra-uterine Cases, &c." has collected several more.

A very valuable paper, describing a case of this kind, written with great accuracy and intelligence by Mr. Mainwaring

---

* Il y á un accident auquel il est toujours impossible à une sage femme, et tres rarement possible à un accoucheur de remedier; c'est celui qui survient à l'occasion de la sortie de l'enfant dans le bas ventre *par l'orifice de la matrice*, ou la rupture de sa substance.

Cette sortie ne se fait qu'en raison de la retroversion et de l'antroversion quoique peu frequentes alors de ce viscere.

L'enfant dans l'un et l'autre cas perit le plus souvent, faute de secours donné à temps eu egard *à la morte subite et presqu'inevitable de la mere.* Cependant la sage femme etant á portée de la prevoir des qu'apres avoir touché la femme en travail et en proie aux plus vives douleurs, elle n'aura pu parvenir à l'orifice de la matrice, elevé alors au-dessus de la symphise des os pubis ou au-dessus de l'os sacrum, elle doit en ce cas demander promptement un accoucheur qui prendra sur lui de se charger des evenements, et sauvera peutetre l'enfant par l'ouverture qu'il pourra pratiquer au bas ventre si elle est indiquée. La conservation de la vie de la mere, dans cette circonstance, est, comme je viens de le dire de la plus grande difficulté, c'est ce qui reste à prouver, mais ce precis n'etant pas fait pour renfermer une dissertation de cette importance et de cette etendue, je la reserve pour mon traité complet d'accouchemens.

Le Bas, Precis de Doctrine sur l'Art d'Accoucheur. Paris, 1780.

† Bartholinus de Insolitis Humani Partus Viis. Cap. 14.

Mainwaring of the Strand, is inserted in the "Transactions of a Society for the Improvement of Medical and Chirurgical Knowledge," vol. 2.

Many of the circumstances of this case coincide in essential particulars with such as occur in retroversions of the uterus, viz.

"Upon an examination per vaginam, a tumour was found in the hollow of the sacrum, occupying its whole extent, and projecting so much forward as nearly to fill the cavity of the pelvis. It seemed to lie between the vagina and the rectum, and was less than two inches within the pelvis, reckoning from the external orifice."

"The os uteri was altered in its shape and situation, being pressed against the bladder and pubes. The cervix uteri was so fixed in its situation as to resist any attempt which was made to move it upwards. From these circumstances the urine was passed with difficulty."

"The shape of the tumour in the hollow of the sacrum (as far as it could be ascertained by examination with the finger) was nearly round, but somewhat flattened upon the anterior part. In breadth it was supposed to be between three and four inches, and in thickness from two to three. It felt moderately firm."

It must not be omitted that Mr. Mainwaring mentions, that "the tumour in the pelvis was lower than that which is found in cases of retroverted uterus; and that the posterior part of the vagina was without the puckering occasioned in that disease by the fundus uteri falling behind the vagina." But I apprehend that these two circumstances cannot be looked upon as proofs of the contrary. The situation of the tumor in the vagina, described above in Mrs. F's case, was much lower than is generally met with in retroversions, and the same may be remarked of Doctor Kelly's case. Indeed, the increased size of the womb at a later period of pregnancy, is sufficient to explain this peculiarity, and likewise the want of puckering in the vagina.

Mr. Mainwaring adds a remark at the conclusion of his very interesting paper, "that the projecting part of the cervix uteri into the vagina, is now * found shorter upon the left side than upon the right," which tends to shew that there is a contraction of the uterus from loss of substance. Now as the tumor which was felt in the vagina evidently communicated with the rectum, may we not infer that this tumor was occasioned by the uterus lying

* Viz. about eighteen months after the discharge of the fœtus.

there there in a retroverted state; and that the contracted state of the womb has been occasioned by the ulcerative process, which could alone have connected it with the rectum?

Mr. Colman, of Norwich, has inserted a case in the second volume of the Medical and Physical Journal, which very much resembles Mr. Mainwaring's, except that the bones of the dissolved fœtus were evacuated through an ulcerated aperture in the vagina instead of the rectum. From the situation of the "os uteri very high above the pubes," I conceive that this was another case of retroverted womb, relieved of its contents by the process of adhesion and ulceration.

Mr. Colman relates, that he was sent for to a woman in labor, attended by a midwife, who had informed her that she would be speedily delivered; but the midwife's promises not being performed in several days, Mr. Colman was called in. On examination, "he found a globular substance very low in the pelvis, which he supposed to be the head of the child, but could not discover the os uteri." He staid with her some time, but as there were no pains he left her, desiring to be sent for again when her labor came on.

About two months afterwards, Mr. Colman hearing that she was not yet delivered, called upon her again, when he found her in the following situation.

"The body had nearly the same appearance as in a natural pregnancy, with an uneveness a little above the os pubis. The whole had not exactly the usual globular form of the impregnated uterus. She had at this time exceeded her reckoning more than two months. I found the child's head pressing down very low, and could not discover the os tincæ in its usual situation, but thought I discovered it above the os pubis."

"On endeavouring to pass the finger towards the sacrum (the usual situation of the mouth of the uterus where it lies high), it could not pass, owing to the vagina obstructing it in every direction backwards. I could pass the finger very high by the pubes, in which situation I found the os uteri as before described. I mentioned my suspicions of its being an extra-uterine fœtus to Mr. Cooper, Surgeon of the 3d Lincolnshire Militia, and requested him to see her with me; he thought it was the os uteri above the pubes, which could not be felt very distinctly as it was situated very high. I examined her again, and concluded it was an extra-uterine fœtus lying between tween the rectum and the womb, pressing the uterus up against and chiefly above the pubes."

It is mentioned that Mr. Rigby, an accoucheur of great experience and reputation, likewise saw and examined this woman; but though he looked upon it as a very extraordinary case, "he was not fully confirmed that it was extra-uterine."

There are so many points of resemblance in the several cases here related or referred to, and the position of the womb, so far as can be gathered from the descriptions, and from the situation of the os tincæ, was so nearly alike in them all, as to afford strong reasons for supposing that they were all of the same nature originally, though they differed widely in their results; there is at least nothing in the opinion of their arising from a retroversion of the womb repugnant to our knowledge of the proceedings of Nature, whereas the theory of extra-uterine fœtuses is inexplicable and unsatisfactory.

It is true, there are proofs enough upon record of extra-uterine conceptions in the ovaria and Fallopian tubes, which are parts so intimately connected with the uterine system, that their productions can scarcely be called extra uterine; and it has been represented that fœtuses more strictly speaking extra-uterine, have been met with in the cavity of the abdomen, when the ovum having accidentally dropped from the ovarium, has there found a nidus, and has advanced nearly, if not quite, to maturity. There is, however, in this hypothesis something so much at variance with all we know of the laws of Nature, and of the animal economy, that it cannot be received without much hesitation.

I mean not however to deny, that fœtuses have been found in the cavity of the abdomen, entirely disengaged from the uterus or its appendages; but think it probable that in such instances, a rupture of the womb, or an ulcerated opening through its parietes, had permitted the escape of the fœtus after it had ceased to live, and not that the conception had advanced to maturity in a part apparently so ill adapted for such a purpose.

It is very justly remarked by Dr. Clarke,* that, "there are so many obstacles to prevent the ovum, when first detached from the ovarium, from acquiring an adhesion in

---

* Transactions of a Society for the Improvement of Medical and Chirurgical Knowledge, vol. ii. p. 15.

in the cavity of the belly, where the intestines, bladder, &c. are from the very nature of their functions in perpetual motion, as to make the possibility of it very doubtful;" he is therefore disposed to conclude, that in all cases supposed to be extra-uterine, the fœtus is really nourished and preserved by the uterus or its appendages; in confirmation of which opinion he adds, "Some years ago, I had the opportunity of seeing a case which had been examined, and was supposed to have been a *true ventral case;* but by a more critical investigation of the case afterwards, it appeared that the cyst containing the ovum was certainly a part of the appendages of the uterus, because it was supplied by the spermatic artery."

It is not necessary to have recourse to the unsatisfactory opinion of an extra-uterine conception to explain the situation of the child between the vagina and rectum. It may be accounted for by supposing it occasioned by a retroversion of the womb; and such an opinion is the more capable of being supported, because it has been demonstrated by more than one well attested fact, that the uterus may remain in a retroverted state till the period of gestation is completed, in which position the child contained in the womb may be easily mistaken for an extra-uterine fœtus.

Though in the instances I have related, the force of the labor-pains was sufficient to replace the womb and exclude their contents through the natural passages, yet no doubt can be entertained that when the uterus is thus unnaturally situated, many circumstances may occur to prevent it from recovering its proper situation. The narrowness of the superior aperture of the pelvis from the projection of the lumbar vertebræ may prove an insurmountable obstacle to the replacement of the womb and to the exclusion of the child through the os uteri and vagina. In such a state, if the pains continue strong, a rupture of the womb would take place, as I conceive happened to Dr. Kelly's and Dr. Mackenzie's patients, and the woman would speedily be carried off; or she might be attacked with fever, or some other cause might be present to occasion a total cessation of the pains, in which event the slow process of inflammation, adhesion and ulceration, would be the consequence; and by such means, if the constitution of the mother could support itself under so great an accumulation of disease, the child would eventually be excluded through the rectum or the posterior part of the vagina.

By such a process we are well assured that several women men have been relieved from their burdens after a pregnancy, sometimes even of many years continuance. In some of these cases the conception has probably been formed either in the tubes or ovaries, and has been excluded from those cavities by means of an abscess and ulcerated aperture, generally near the navel.* No doubt can be entertained of the possibility that the same process may take place in the retroverted womb, and whenever the abscess has opened into the rectum or vagina, it is probable that the situation of the fœtus between those two passages will be often found to have been occasioned by that position of the uterus.

If this conjecture be correct, it will explain some peculiarities in the history of extra-uterine fœtuses hitherto unaccountable, particularly the enlargement of the womb and the access of uterine pains at the termination of the period of pregnancy. It has been observed in every case of fœtus carried in the abdomen beyond the period of nine months, that about or before the usual time of reckoning, the pains of labor have regularly come on, and strong efforts have been made by the womb as if to expel the child. It is difficult to assign any reason for these contractions of the womb, if the fœtus have no connection with but be entirely distinct from that organ; if, however, the fœtus be lodged in the retroverted womb, or even in any of the appendages of the uterus, the occurrence of labor pains will excite less astonishment.

It is not however my intention to enter at large into a discussion of the reality of extra-uterine conceptions; my motive in publishing these few observations is principally, by demonstrating that the womb may occasionally be found retroverted at the end of nine months, to put practitioners of midwifery on their guard, should they meet with an instance of the kind; and I have been the more readily induced to do this, because it has been recommended by some accoucheurs of deserved celebrity,†

---

* May not this unfortunate situation have been occasionally produced by a kind of hernia or partial rupture of the uterus from a protrusion of its inner coat through its external fibres, forming a pouch in which the fœtus has been lodged?

† Among others may be enumerated Dr. Kelly and Dr. Mackenzie, already quoted, men of much experience and judgment; and Dr. Hull, the zealous defender of the Cæsarean section, who, in support of his recommendation, copies from Sabatier's Traité de Medecine Operatoire, the case of a child extracted by means of an incision through the vagina from (I conceive) the retroverted womb, with safety, as it appears, to the mother. Observations on Mr. Simmons's Detection, &c. part ii. p. 223—233.

when a tumor containing a fœtus shall be discovered by the touch, situated between the vagina and rectum, that an incision should be made through the posterior part of the vagina, as the most probable means of affording the patient relief.

It would, I am sensible, be advancing too much to assert that such an operation can never be necessary, because cases may happen in which the only chance of saving the mother's life may consist in freeing her from the fœtus by means of an incision; but surely the operation ought not to be advised, till it is ascertained that the child is not retained in the retroverted womb, and of course that the pains of labor cannot effect the delivery; and even then, with so many examples before us of the exclusion of putrid and dissolved fœtuses safely by the ulcerative process, it may be doubted whether we ought not rather to apply our endeavours towards supporting the strength of the mother, and conducting her through that process with as little suffering as the art of medicine can procure.

Wounds of the uterus have been proved by so much experience to be almost constantly fatal in this country, that nothing but the most urgent necessity can justify the accoucheur in making an artificial aperture into that viscus; the recommendation therefore of making an incision through the vagina in cases where the child is felt situated between the vagina and rectum, ought not to be implicitly followed, for should that position of the child be occasioned by a retroversion of the womb, the most fatal consequences might ensue from such an operation, while the favorable termination, (at least with regard to the mothers) which attended the cases related in the former part of this communication, may encourage us to wait patiently for the gradual operations of nature, without irritating the vagina or attempting any means of exciting the premature exclusion of the fœtus; for, as it has been very truly said by Bartholine after Cicero,

> Naturæ solertiam nulla ars, nulla manus, nemo opifex consequi potest imitando. De Insolitis Humani Partus Viis.

This precept, therefore, cannot be too strongly inculcated, that Nature (except in a few cases) is fully sufficient for her own work, and that women are not in general injured by a long continuance of labor pains, provided their spirits are not hurried, nor their blood inflamed by injudicious attempts to give assistance, and by the improper use of cordials and stimulants.

